# Genetic diversity and population structure of *Glossina morsitans morsitans* in the active foci of human African trypanosomiasis in Zambia and Malawi

**DOI:** 10.1371/journal.pntd.0007568

**Published:** 2019-07-25

**Authors:** Yukiko Nakamura, Junya Yamagishi, Kyoko Hayashida, Naoki Osada, Elisha Chatanga, Cornelius Mweempwa, Kalinga Chilongo, John Chisi, Janelisa Musaya, Noboru Inoue, Boniface Namangala, Chihiro Sugimoto

**Affiliations:** 1 Division of Collaboration and Education, Research Center for Zoonosis Control, Hokkaido University, Sapporo, Hokkaido, Japan; 2 Global Station for Zoonosis Control, GI-CORE, Hokkaido University, Sapporo, Hokkaido, Japan; 3 Graduate School of Information Science and Technology, Hokkaido University, Sapporo, Hokkaido, Japan; 4 Global Station for Big Data and Cybersecurity, GI-CoRE, Hokkaido University, Sapporo, Hokkaido, Japan; 5 Laboratory of Parasitology, Graduate School of Infectious Diseases, Hokkaido University, Sapporo, Hokkaido, Japan; 6 Department of Veterinary Medicine, Lilongwe University of Agriculture and Natural Resources, Lilongwe, Malawi; 7 Department of Veterinary Services, Tsetse and Trypanosomiasis Control Unit, Ministry of Fisheries and Livestock, Lusaka, Zambia; 8 Department of Basic Medical Science, College of Medicine, University of Malawi, Blantyre, Malawi; 9 Department of Pathology, College of Medicine, University of Malawi, Blantyre, Malawi; 10 Obihiro University of Agriculture and Veterinary Medicine, Inada, Obihiro, Hokkaido, Japan; 11 Department of Paraclinical Studies, School of Veterinary Medicine, University of Zambia, Lusaka, Zambia; Yale School of Public Health, UNITED STATES

## Abstract

The tsetse fly, *Glossina morsitans morsitans*, is a significant problem in Zambia and Malawi. It is the vector for the human infective parasite *Trypanosoma brucei rhodesiense*, which causes human African trypanosomiasis, and various *Trypanosoma* species, which cause African animal trypanosomiasis. Understanding the genetic diversity and population structure of *G*. *m*. *morsitans* is the basis of elucidating the connectivity of the tsetse fly populations, information that is essential in implementing successful tsetse fly control activities. This study conducted a population genetic study using partial mitochondrial cytochrome oxidase gene 1 (CO1) and 10 microsatellite loci to investigate the genetic diversity and population structure of *G*. *m*. *morsitans* captured in the major HAT foci in Zambia and Malawi. We have included 108 and 99 *G*. *m*. *morsitans* samples for CO1 and microsatellite analyses respectively. Our results suggest the presence of two different genetic clusters of *G*. *m*. *morsitans*, existing East and West of the escarpment of the Great Rift Valley. We have also revealed genetic similarity between the *G*. *m*. *morsitans* in Kasungu National Park and those in the Luangwa river basin in Zambia, indicating that this population should also be included in this historical tsetse belt. Although further investigation is necessary to illustrate the whole picture in East and Southern Africa, this study has extended our knowledge of the population structure of *G*. *m*. *morsitans* in Southern Africa.

## Author summary

Techniques from population genetics have been widely applied to assess problems in evolutionary biology, computational biology, wildlife conservation, animal breeding, etc. In this study, we used population genetics approaches to elucidate the genetic population structure of the tsetse fly *Glossina morsitans morsitans*, an important vector of the *Trypanosoma* parasite. Our aim was to identify the optimal range of areas in which to implement effective tsetse fly control programs. We focused on five areas of active human African trypanosomiasis foci in Zambia and Malawi and used both mitochondrial and nuclear markers for population genetics analyses. High levels of genetic differentiation were observed between *G*. *m*. *morsitans* in Nkhotakota Wildlife Reserve, Malawi, and those at the other locations included in this study. Nkhotakota Wildlife Reserve lies on the eastern side of the escarpment of the Great Rift Valley at an altitude that is at the biological limit of *G*. *m*. *morsitans*, thereby functioning as a geographical barrier restricting gene flow from the *G*. *m*. *morsitans* on the western side of the escarpment. The western side of the escarpment is part of a historically-known tsetse belt that resides within the Luangwa river basin. We have demonstrated that a previously undescribed population of *G*. *m*. *morsitans* in Kasungu National Park, Malawi, should be included in this historical tsetse belt.

## Introduction

Tsetse flies (*Glossina spp*.) are the vector for the *Trypanosoma* parasites that cause human African trypanosomiasis (HAT) and African animal trypanosomiasis (AAT). Both diseases present a significant burden in terms of public health and economy in sub-Saharan Africa [1,2]. Control of both diseases are difficult, mainly due to the presence of wild and domestic animal reservoirs, the lack of prophylactic drugs and vaccines, and the high cost and severe side effects of available drugs. Therefore, tsetse fly control, in combination with chemotherapeutic methods, is still the most theoretically desirable method for controlling both AAT and HAT [3,4]. To conduct effective tsetse fly control programs, it is necessary to identify the extent of the tsetse fly distribution and its connectivity with residing populations. Several population genetics studies have been successful in identifying population structure and the extent to which the discrete populations are connected by dispersal and migration in several tsetse-infested African countries [5–8].

Zambia and Malawi, along with Mozambique and Zimbabwe, lie within the “common fly belt” in Southern Africa [9]. Tsetse flies found in these areas include three subspecies or species of the Morsitans group (subgenera *Glossina* Wiedemann) of tsetse flies: *G*. *morsitans morsitans*, *G*. *morsitans centralis*, and G. *pallidipes*; one species from the Fusca group (subgenera *Austenina* Townsend); *G*. *brevipalpis;* and one species from the Palpalis group (subgenera *Nemorhina* Robineau-Desvoidy); G. *fuscipes* [10]. Among these species of tsetse flies, *G*. *m*. *morsitans* and *G*. *pallidipes* are the major vectors of AAT and HAT in these countries. The prevalence of human infective *T*. *b*. *rhodesiense*, as assessed by the presence of the *T*. *b*. *rhodesiense*-specific human serum resistance-associated gene, was highest in *G*. *m*. *morsitans* [11]. *G*. *m*. *morsitans* inhabits savanna woodlands, and its distribution has been mapped primarily by determining the abundance of mammal hosts, and the analyses of tsetse habitat using geographical information systems and remotely-sensed satellite data [12,13]. The *G*. *m*. *morsitans* populations in East and Southern Africa are distributed across Tanzania, Mozambique, Zimbabwe, Zambia and Malawi [14] and constitute four allopatric belts [13]. The genetic diversity and population structure of *G*. *m*. *morsitans* in two of the four allopatric belts has been explored using mitochondrial and microsatellite markers, and significant differences have been identified among five populations from Zambia, Mozambique and Zimbabwe [15,16]. Further research involving four populations from Tanzania has also revealed high differentiation and low rates of gene flow, suggesting that the discontinuous distribution of the populations leads to genetic drift, overwhelming gene flow [17]. Although it has been suggested that further sampling from other allopatric belts would increase our understanding of the breeding structure of *G*. *m*. *morsitans* in this region [15], there has, to our knowledge, been no research conducted that includes *G*. *m*. *morsitans* populations from Malawi.

Zambia and Malawi, two countries in South-East Africa with a significant HAT burden, report fewer than100 new cases annually [1]. The major HAT loci are the Lower Zambezi region and the Luangwa valley in eastern Zambia [18], and Kasungu National Park and Nhkotakota Wildlife Reserve in Malawi [19]. The two countries share foci in the Northern region: The Chama district of Zambia and the Rumphi district of Malawi. Therefore, both countries should be included when assessing questions related to HAT. To investigate the genetic diversity and population structure of *G*. *m*. *morsitans* in the major HAT loci in Zambia and Malawi, which data are fundamental for vector control, we conducted a population genetics study using a 407-bp region of mitochondrial cytochrome oxidase 1 gene (CO1) with 10 microsatellite loci as genetic markers.

## Materials and methods

### Sample collection and DNA extraction

**Fig 1:**
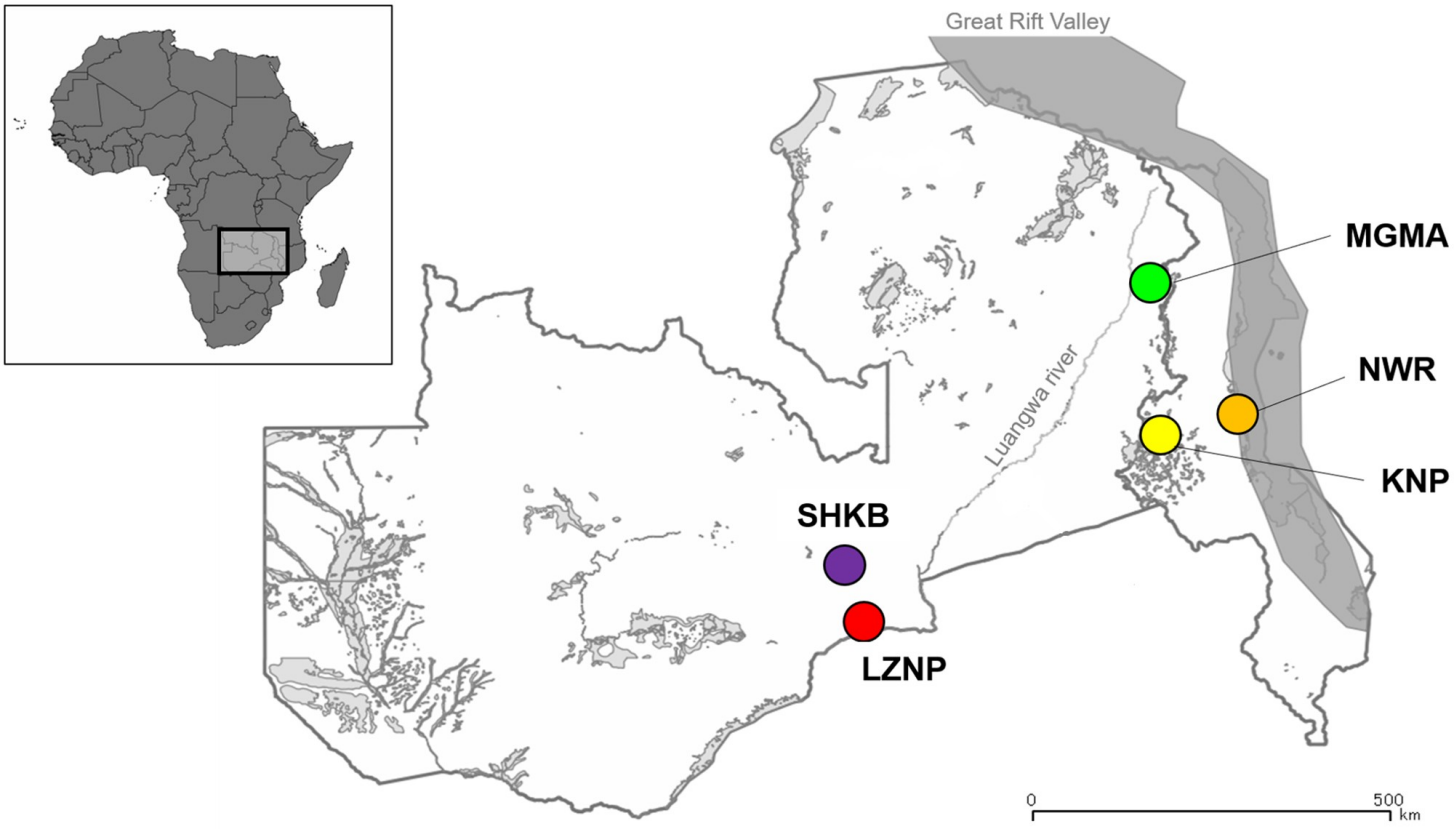
Map of Zambia and Malawi showing sampling locations for *Glossina morsitans morsitans*. Markers indicate the five sampling locations for the *G*. *m*. *morsitans* populations included in this study. The full names of the location codes can be found in Table 1. The layers were obtained from MapCruzin.com (https://mapcruzin.com/), and the figure was created using QGIS v3.0 (https://qgis.org/en/site/).

*G*. *m*. *morsitans* were collected from three locations in Zambia, and two locations in Malawi (Fig 1). The three sampling locations in Zambia include the Lower Zambezi National Park (LZNP, S15° 37.577, E29° 36.132), Shikabeta (SHKB, S14° 57.208, E29° 49.924), and the Musalangu Game Management Area (MGMA, S11° 09.807, E33° 24.224). Sampling within the national parks and GMAs was conducted with permission from the Zambian Wildlife Authority (ZAWA). The two sampling locations in Malawi include the Kasungu National Park (KNP, S13° 01.289, E33° 08.517) and the Nkhotakota Wildlife Reserve (NWR, S12° 52.212, E34° 08.283), in which sampling was conducted with permission from the Department of National Parks and Wildlife (DNPW) of Malawi. Samples were collected between May 2012 and February 2018. At each sampling location, flies were captured when driving down roads while deploying mobile tsetse traps attached to the rear end of the car. Each drive was around one kilometer, a distance which is within the average lifetime dispersal of *G*. *morsitans* [20]. The captured flies were inspected using microscopy for morphological identification of *G*. *m*. *morsitans* [21] and sexing. Apparently pregnant females were not included in the analysis. The flies were then put into separate 2 mL sample tubes with silica beads to dry. The dried flies were transferred into new tubes with beads, and were smashed using a Beads Cell Disrupter (Micro Smash MS-100, Tomy, Japan) at 3,000 rpm for 45 s. DNA was extracted using a modified protocol with the DNA Isolation Kit for Mammalian Blood (Roche, Switzerland). Briefly, 330 μl of white cell lysis buffer was added directly into each tube, vortexed, and heated at 37°C for 30 min. Then 170 μl of protein precipitation solution was added, vortexed thoroughly, and centrifuged at 15,000 rpm for 20 min. DNA was precipitated by the addition of ethanol. Extracted DNA was stored at −30°C until further use.

### CO1 amplification, sequencing, and analyses

We generated new primers (Forward: 5′–CTT TAC CTG TAT TAG CCG GAG C–3′, Reverse: 5′–ACT CCT GTT AAA CCT CCT ACT G–3′) for the amplification of a 477-bp fragment of the mitochondrial CO1 gene. Reactions contained 0.25 *μ*l (1–10 ng) of template DNA, 5 *μ*l Ampdirect Plus (Shimadzu Corp.), 0.05 *μ*l BioTaq HS DNA Polymerase (Bioline), 0.5 *μ*l (10 mM) primers, and 3.7 *μ*l of water for a total volume of 10 *μ*l. Amplification included an initial denaturation step at 95°C for 10 min, followed by 94°C for 30 s of denaturation, 30 cycles each for 30 s at 60°C for annealing, 1 min at 72°C for extension, and a final extension step at 72°C for 7 min. The PCR products were purified using ExoSAP-IT (Applied Biosystems), following the manufacturer’s protocol. Forward and reverse strands were sequenced with ABI 3130/3500xl sequencers (Applied Biosystems). The chromatograms were visually inspected, and poor-quality data were trimmed using ApE Plasmid Editor v2.0.51 (M. Wayne Davis, Univ. Utah, USA). Both forward and reverse sequences were used to create a consensus sequence for each sample, and multiple sequences were aligned using online MAFFT v7 [22], resulting in 108 fully aligned 407-bp sequence fragments.

The summary statistics calculated were: the average number of nucleotide differences between individuals within locations (*Nd*); the average nucleotide diversity within locations (*π*); the number of haplotypes within locations (*H*); and haplotype diversity within locations (*Hd*). the summary statistics and the mismatch distribution of pairwise differences were analyzed using DnaSP v6.11.01 (Julio Rozas, Universitat de Barcelona, Spain) [23]. Neutrality tests (Tajima’s *D*: based on the difference between expected segregating sites, and Fu’s *F*_*s*_: based on the degree of excess of rare alleles), population pairwise *ϕ*_ST_ (an analogue of *F*_ST_, which estimates the deviation from random mating among demes) and the genetic diversity within and among populations evaluated by the analysis of molecular variance (AMOVA) using the haplotype frequencies distance method, were analyzed using ARLEQUIN v3.5.2.2 [24,25]. Bonferroni correction was used for multiple-comparison correction of population pairwise comparisons.

To infer and visualize the evolutionary relationships of the haplotypes, we constructed a median-joining haplotype network using POPART v1.7 [26].

### Microsatellite amplification and marker validation

We used 12 autosomal microsatellite loci, which have been described in other studies [27–30]. Amplifications were performed using fluorescently-labeled forward primers (FAM, VIC, NED, and PET) in a reaction volume of 10 *μ*l, containing 1 *μ*l template DNA, 0.05 *μ*l Multiplex PCR mix 1 and 5.0 *μ*l Multiplex PCR mix 2 (Multiplex PCR Assay Kit, Takara), 0.2 *μ*l (10 mM) primers, and 3.55 *μ*l of water. Amplification included an initial denaturation step at 94°C for 1 min, followed by 94°C for 30 s of denaturation, 30 cycles each of 90 s at 57°C for annealing, 90 s at 72°C for extension, and a final extension step at 72°C for 10 min. PCR products were diluted from ×1 to ×20 according to the thickness of the band from the electrophoresis results, and four different fluorescence samples were pooled to be genotyped on an ABI 3130 sequencer. Alleles were scored using the software Peak Scanner v1.5 (Applied Biosystems) and the scored peaks were manually edited. Micro-Checker v2.2.3 was used to check for null alleles, and two loci were dropped due to the presence of null alleles [31], resulting in using 10 loci for further analyses.

### Microsatellite analyses

MSA v4.05 [32] was used to generate a *genepop* file, which was used in GENEPOP v4.7 [33] to test for deviation from Hardy-Weinberg equilibrium and linkage disequilibrium (LD) using the Markov chain method with 10,000 dememorizations, 1,000 batches and 10,000 iterations per batch [34]. Genetic diversity indices including the mean number of alleles (*N*_*A*_), allelic size range (*A*_*S*_, the range in nucleotide length among microsatellite alleles), expected heterozygosity among polymorphic loci (*H*_*E*_), observed heterozygosity among polymorphic loci (*H*_*O*_), estimation of the inbreeding coefficients (*F*_IS_, estimation of the deviation from random mating within demes), and population pairwise *F*_ST_ based on number of different alleles were calculated using ARLEQUIN v3.5.2.2. The genetic diversity within and among populations were calculated by AMOVA analysis as implemented in ARLEQUIN v3.5.2.2 [25]. Bonferroni correction was used for multiple-comparison correction of the population pairwise comparison.

The genetic structure of *G*. *m*. *morsitans* was determined using the Bayesian clustering method used in STRUCTURE v2.3.4 [35]. Ten replicate runs for each K = 1–10 were carried out with a burn-in length of 20,000 followed by 200,000 iterations. The most likely value of K was determined using the Evanno method [36] implemented in STRUCTURE HARVESTER v0.6.94 [37]. The replicates for the most likely K were aligned using CLUMPP v1.1.2 [38], and the aligned cluster assignments were visualized using DISTRUCT v1.1 [39].

The effective population size (*Ne*), including both female and male samples, was estimated for each population using the LD method in NeEstimator v2.1 [40], and tests for population bottleneck occurrence was conducted using the two-phase mutation (TPM) model implemented in BOTTLENECK v1.2.02 [41], as recommended for microsatellite loci [42]. Significance was assessed using Wilcoxon’s signed-rank test. Tests for recent bottleneck events were carried out using a mode-shift indicator of allele frequency distributions [43].

### Accession list

CO1 sequences have been uploaded to DDBJ:

**Table:** 

Accession number	EntryID
LC455935	5c2eb83cd25b2c93592674e5.Hap_1
LC455936	5c2eb83cd25b2c93592674e5.Hap_2
LC455937	5c2eb83cd25b2c93592674e5.Hap_3
LC455938	5c2eb83cd25b2c93592674e5.Hap_4
LC455939	5c2eb83cd25b2c93592674e5.Hap_5
LC455940	5c2eb83cd25b2c93592674e5.Hap_6
LC455941	5c2eb83cd25b2c93592674e5.Hap_7
LC455942	5c2eb83cd25b2c93592674e5.Hap_8
LC455943	5c2eb83cd25b2c93592674e5.Hap_9
LC455944	5c2eb83cd25b2c93592674e5.Hap_10
LC455945	5c2eb83cd25b2c93592674e5.Hap_11
LC455946	5c2eb83cd25b2c93592674e5.Hap_12
LC455947	5c2eb83cd25b2c93592674e5.Hap_13
LC455948	5c2eb83cd25b2c93592674e5.Hap_14
LC455949	5c2eb83cd25b2c93592674e5.Hap_15
LC455950	5c2eb83cd25b2c93592674e5.Hap_16
LC455951	5c2eb83cd25b2c93592674e5.Hap_17
LC458946	5c471811cb371656035ec273.Hap_1_Malawi
LC458947	5c471811cb371656035ec273.Hap_3_Malawi
LC458948	5c471811cb371656035ec273.Hap_4_Malawi
LC458949	5c471811cb371656035ec273.Hap_5_Malawi
LC458950	5c471811cb371656035ec273.Hap_6_Malawi
LC458951	5c471811cb371656035ec273.Hap_8_Malawi
LC458952	5c471811cb371656035ec273.Hap_9_Malawi
LC458953	5c471811cb371656035ec273.Hap_13_Malawi

Microsatellite genotype data available from the Dryad Digital Repository: **doi:**10.5061/dryad.122hs54.

## Results

### CO1 genetic diversity and haplotype diversity

**Fig 2:**
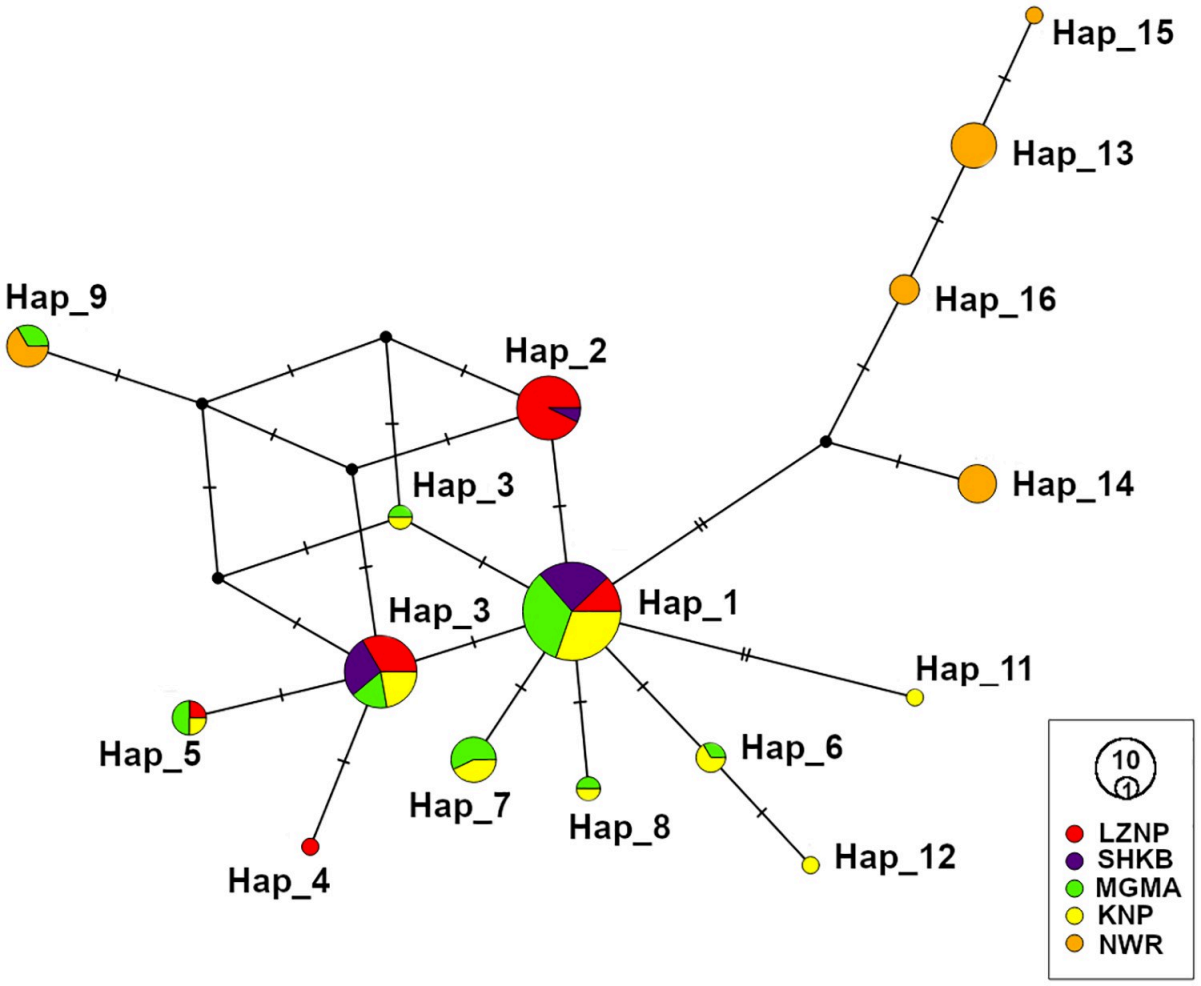
Median-joining haplotype network of CO1 sequences. The median-joining network was constructed using 108 CO1 sequences and was visualized using POPART v1.7. Each circle represents a haplotype, and the size of a circle is proportional to the number of sequences assigned to that haplotype. The location from which the sequence was obtained is indicated by color in the legend. The number of hatch marks indicate the number of nucleotide differences that separate the haplotypes. A black dot represents an intermediate missing haplotype.

Analysis of the 407-bp fragment of the CO1 gene of 108 individual *G*. *m*. *morsitans* flies from five locations resulted in the identification of 16 haplotypes, Hap_1 to Hap_16 (Fig 2, Table 1). The number of haplotypes found within each sampling location in Zambia and Malawi varied from three to nine. Haplotype diversity (*Hd*) in these locations were generally high, ranging from 0.582 in SHKB to 0.801 in KNP. In contrast, nucleotide diversity (*π*) was low, ranging from 0.002 in SHKB to 0.007 in NWR. The most common haplotype, Hap_1, was found in 33 individuals (30.6%) from all sampling locations except NWR, indicating a common ancestry of the *G*. *m*. *morsitans* from four locations (S1 Table). The second most common haplotype Hap_3 was found in 18 individuals (16.7%) from four sampling locations. Seven other haplotypes occurred in two or three sampling locations, and the other seven haplotypes were unique to specific localities and were dubbed “private” haplotypes. Among the private haplotypes, four were singletons, where the haplotype was found in only a single individual. Interestingly, none of the haplotypes were shared between KNP and NWR despite their relatively close geographic distance. The only haplotype that was not unique to NWR was Hap_9 which occurred in both NWR and MGMA. KNP shared many common haplotypes with the Zambian locations and seems to be a part of the major haplogroup. The other four haplotypes (Hap_13, Hap_14, Hap_15, and Hap_16) observed in NWR formed a haplogroup that was separated from the major haplogroup by an inferred missing haplotype (Fig 2).

**Table 1:** *Glossina morsitans morsitans* sampling sites and genetic diversity statistics for CO1 sequences and microsatellite fragments.

Sampling locations	Location code	Sampling date	CO1					Microsatellite					
			*n*	*Nd*	*π*	*H*	*Hd*	*N*	*N*_*A*_	*A*_*S*_	*H*_*E*_	*H*_*O*_	*F*_*IS*_
Lower Zambezi National Park	LZNP	2012 May	25	1.213	0.003	5	0.670	40	10.1	36.5	0.835	0.9	−0.086
Shikabeta	SHKB	2017 Oct	14	0.637	0.002	3	0.582	38	8.8	34.9	0.768	0.890	−0.163
Musalangu Game Management Area	MGMA	2017 Oct	25	1.540	0.004	8	0.780	40	9.8	36.3	0.815	0.860	−0.057
Kasungu National Park	KNP	2018 Mar	24	1.301	0.003	9	0.801	40	8.8	41.8	0.798	0.935	−0.177
Nkhotakota Wildlife Reserve	NWR	2018 Feb	20	2.868	0.007	5	0.790	40	9.0	40.6	0.817	0.905	−0.111
Total			108			16		198					
Mean				1.512	0.004		0.725		9.3	38.0	0.807	0.898	−0.119

The location code for each sampling location is indicated. For CO1: *n* = number of individuals, *Nd* = average number of nucleotide differences within locations, *π* = average nucleotide diversity within locations, *H* = number of haplotypes, *Hd* = haplotype diversity. For microsatellite: *N* = number of gene copies, *N*_A_ = mean number of alleles, *A*_S_ = allelic size range, *H*_E_ = expected heterozygosity among polymorphic loci, *H*_O_ = observed heterozygosity among polymorphic loci, *F*_IS_ = Fisher’s inbreeding coefficient.

We tested for neutrality using both Tajima’s *D* and Fu’s *F*_S_ test. As a result, KNP showed significant *p* values for both Tajima’s *D* and Fu’s *F*_S_ (*p* < 0.05, *p* < 0.02 respectively), suggesting the effects of either positive selection or population expansion in KNP (Table 2). From the mismatch distribution of pairwise differences, the observed distribution had a bimodal distribution (S1 Fig; Raggedness index: 0.1088, Mean Absolute Error: 0.6533).

**Table 2:** Neutrality test results.

Statistics	LZNP	SHKB	MGMA	KNP	NWR	Mean	s.d.
Sample size	25	14	25	24	20	21.6	4.722
Tajima’ *D*	−0.40	0.037	−0.867	−1.509	0.917	−0.364	0.917
Tajima’s *D p*-value	0.443	0.655	0.227	**0.041**	0.846	0.442	0.322
No. of alleles (unchecked)	5	3	8	9	5	6.0	2.449
Theta pi	1.213	0.637	1.540	1.301	2.868	1.512	0.828
Exp. no. of alleles	4.270	2.588	4.907	4.396	6.420	4.516	1.376
Fu’s *Fs*	−0.335	−0.040	−2.610	−4.683	1.773	−1.179	2.502
*Fs p*-value	0.420	0.350	0.045	**0.004**	0.841	0.332	0.338

Statistically significant values at Tajima’s *D* (p < 0.05) and Fu’s *F*s (*p* < 0.02) are indicated in bold.

The AMOVA analysis of the five locations indicated that the overall genetic variation within populations was larger (84.29%) than the variation among populations (15.71%) (Table 3), suggesting little genetic structure. However, population pairwise *ϕ*_ST_ values showed significantly different values after Bonferroni correction (*p* < 0.05) between NWR and other locations, with values ranging from 0.203 to 0.307. All comparisons between LZNP and the other locations showed significant *ϕ*_ST_ values, ranging from 0.177 to 0.273 (Table 4).

**Table 3:** Results of AMOVA on CO1 and microsatellites.

	source of variation	d.f.	sum of squares	variance components	percentage of variation	*p*-values
CO1	among populations	5	8.431	0.069	15.71	<0.001
	within populations	111	41.005	0.369	84.29	
	Total	116	49.436	0.438		
Microsatellite	among populations	4	32.256	0.114	2.76	<1.000
	among individuals within populations	94	333.133	-0.470	-11.39	<0.001
	within individuals	99	444	4.485	108.63	<1.000
	Total	197	809.389	4.129		

AMOVA was conducted using ARLEQUIN v3.5.2.2, on 108 CO1 samples and 99 microsatellite samples from five sampling locations. Significance was estimated using 1023 random permutations.

**Table 4:** Results of population pairwise comparison among five sampling locations.

	LZNP	SHKB	MGMA	KNP	NWR
LZNP		**0.015**	0.005	0.009	**0.024**
SHKB	**0.198**		0.002	0.002	**0.075**
MGMA	**0.195**	0.029		0.013	**0.032**
KNP	**0.177**	0.010	−0.031		**0.065**
NWR	**0.273**	**0.307**	**0.203**	**0.205**	

The lower diagonal in Table 4 shows the results of population pairwise *ϕ*_ST_ comparisons among 108 CO1 sequences based on haplotype differences. The upper diagonal represents the results of population pairwise *F*_ST_ comparisons based on the number of different alleles among 99 microsatellite samples. Numbers in bold show statistically significant values at the *p* < 0.05 significance level, after Bonferroni correction.

### Microsatellite genetic diversity and population structure

A total of 99 *G*. *m*. *morsitans* samples were genotyped at 10 microsatellite loci. None of the microsatellite loci pairs showed significant results of the LD tests (S2 Table). *N*_*A*_ was highest in LZNP (10.1) and lowest in SHKB and KNP (8.8) in the five locations (Table 1). The lowest *H*_*E*_ was observed in SHKB (0.768), and the highest was observed in LZNP (0.835).

The percentage of variation within individuals was highest (108.63%) compared to the percentage of variation among populations (2.76%) and among individuals within populations (−11.39%) in the AMOVA analysis (Table 3). The *F*_ST_ estimate among locations was 0.028 (*p* < 0.001). All pairwise comparisons between NWR and other locations were statistically significant (*p* > 0.05), with *F*_ST_ values after Bonferroni correction ranging from 0.024 (NWR vs. LZNP) to 0.075 (NWR vs. SHKB) (Table 4), indicating small to moderate genetic distance according to Wright’s criteria [44]. The other pairwise comparison (SHKB vs. LZNP) which showed statistical significance at *p* > 0.05 had a value of 0.015, indicating low genetic distance [44].

**Fig 3:**
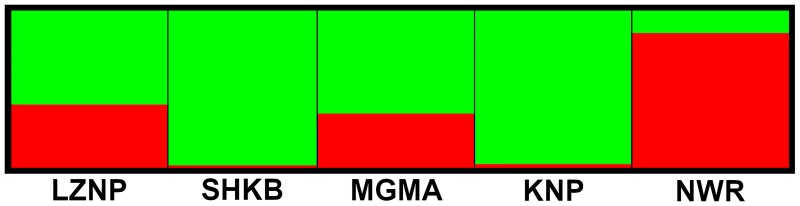
STRUCTURE results. Structure plot for K = 2 inferred populations, based on individuals from all five locations.

Including all five locations in the STRUCTURE analysis, the Evanno method resulted in the identification of two genetic clusters (S2 Fig). NWR was the only location in which the majority of the genetic cluster is shown as red, whereas the majority of the clusters in the other locations were green. This observation indicates the presence of high genetic divergence between NWR and the other four locations (Fig 3).

*Ne* was estimated using the LD method. SHKB and KNP had 95% confidence intervals which included infinity. LZNP, MGMA, and NWR showed *Ne* of 121.1 (95%CI 65.0–606.2), 133.5 (95%CI 65.7–2763.0), and 32.1 (95% CI 24.2–45.5), respectively. NWR had relatively low *Ne* values compared to the other locations (Table 5). None of the locations were positive using BOTTLENECK analysis under the TPM model (Table 5). All five populations were approximated to have the expected normal L-shaped allele frequency distributions, and are therefore likely to be near mutation-drift equilibrium [43] (Table 5).

**Table 5:** Effective population size estimates and tests for bottlenecks.

	Sample size	*Ne*	95% Cl	TPM *p*-value	Mode-shift
LZNP	20	121.1	65.0–606.2	0.322	Normal L-shaped
SHKB	19	infinite	183.8–infinite	0.131	Normal L-shaped
MGMA	20	133.5	65.7–2763.0	0.160	Normal L-shaped
KNP	20	infinite	191.7–infinite	0.193	Normal L-shaped
NWR	20	32.1	24.2–45.5	0.625	Normal L-shaped

The effective population size estimates (*Ne)* was calculated using the LD model. Bottleneck tests were assessed using the two-phase mutation model (TPM) and shown as *p*-values, based on Mann–Whitney U test. Bold numbers indicate significance level of *p* < 0.05.

## Discussion

### Population structure

Our haplotype network analysis of the CO1 sequences and microsatellite STRUCTURE analyses revealed high differentiation between *G*. *m*. *morsitans* from NWR from other locations (Figs 2 and 3). The pairwise population comparison of mitochondrial CO1 (*ϕ*_ST_, lower diagonal in Table 4) also suggested high genetic distance between the flies in NWR and the flies from other locations. The pairwise population comparison of microsatellite alleles (*F*_ST_, upper diagonal in Table 4) also showed significant *F*_ST_ values between NWR and the other locations but had relatively low values, ranging from 0.024 to 0.075. Pairwise *F*_ST_ values ranged between 0.027 and 0.161 in a previous study of *G*. *m*. *morsitans* among one population from Zambia, three populations from Zimbabwe, one population from Mozambique, and four populations from Tanzania, and pairwise genetic differences were considered to be high when the *F*_ST_ value exceeded 0.03 [17]. When we used the same criteria, the overall pairwise genetic differences were high, except for the comparisons of LZNP with SHKB (*F*_ST_ = 0.015) and LZNP with NWR (*F*_ST_ = 0.024). The discrepancy between CO1 and microsatellite pairwise comparisons may be due to differences in the level of differentiation between mitochondrial and nuclear genomes. The mitochondrial genome is known to reach genetic-drift equilibrium earlier than the nuclear genome, due to the smaller effective population size of the mitochondrial genome. These results suggest that there is limited gene flow between *G*. *m*. *morsitans* in NWR from the same subspecies found in other locations, a finding that corresponds well with the estimation of *G*. *m*. *morsitans* distribution conducted in previous studies [12,13,17]. NWR is adjacent to Lake Malawi, a southern constituent of the Great Rift Valley. This area extends from a 1638 m high escarpment in the West and stretches East up to a narrow plain beside Lake Malawi, which has an altitude of around 500 m. This escarpment is a potential geographical barrier preventing gene flow between NWR and other locations studied, since the upper altitudinal limit for the survival of tsetse flies is known to be in a range of 1600 m to 2200 m [45,46], which underlying variable is temperature [13].

LZNP also exhibited statistically significant *ϕ*_ST_ values compared with other locations (Table 4). However, since there are five to six years-interval between the sampling date between LZNP and the other locations, these results may be due to temporal flux over this interval. LZNP, SHKB, and MGMA are all included in the Luangwa river basin (Luangwa tsetse belt), as illustrated in the *G*. *m*. *morsitans* distribution maps [12–14]. However, there has been no identification of *G*. *m*. *morsitans* around KNP in any previously reported maps. According to our results, KNP did not show evidence of genetic differentiation from the other locations in the Luangwa tsetse belt, and should therefore be included in this historical tsetse belt.

### Population size changes

High *Hd* and low *π* were inferred from the results of the CO1 analysis (Table 1). This pattern of high *Hd* and low *π* is consistent with observations of other *Glossina* species in Uganda and Kenya [47,48], and suggests that the populations have experienced a significant population decline resulting in the loss of genetic diversity and subsequently diverged into different haplotypes as the population size recovered. The tsetse fly population in Southern Africa is known to have experienced severe decreases due to a rinderpest epizootic in the 1890s, leading to an up to 90% decline in the size of wildlife populations [49]. Since the major blood meal source of *G*. *m*. *morsitans* are wildlife, this event is possibly the cause of the historic loss of genetic diversity in southern African countries such as Zambia and Malawi. Subsequently, the *G*. *m*. *morsitans* population may have expanded as wildlife recovered from the rinderpest epidemic [50]. This hypothesis, that the high *Hd* and low *π* observed in this study reflects the state of recovery from the rinderpest epidemic, remains speculative since the BOTTLENECK analysis did not detect a positive event in any of the locations included in this study (Table 5). It is likely that the sample size used in this study was insufficient to detect a bottleneck event 120 years ago, or that sufficient generations have passed between the presumed bottleneck event and the sampling generation to allow re-establishment of a mutation-drift equilibrium. This method is known to be able to detect bottleneck events up to 40–80 generations in the past [17,43], and 120 years is approximately 973 tsetse fly generations (the generations were estimated by using a lifecycle of 45 days per fly) [51]. Relatively large *Ne* have been estimated for SHKB and KNP, both including infinity within the 95% confidence interval. Population expansion or selection was suggested for KNP, since this site showed statistically significant negative values in both Tajima’s *D* and Fu’s *F*_s_ (Table 2). Both tests will test the deviation from equilibrium expectations based on the infinite-site model without recombination. Negative values suggest recent population expansion, or decrease in genetic variation due to positive selection. The observed mismatch distribution of pairwise differences had a bimodal distribution (S1 Fig) with low fitness to the estimated allele frequency under a population expansion model (Raggedness index: 0.1088, Mean Absolute Error: 0.6533). This indicates low possibility of population expansion [52]. However, in order to differentiate population expansion and selection, factors such as the evenness of the distribution of mutation across the whole genome and the ratio between nonsynonymous and synonymous mutations must be explored [53]. Therefore, further investigations are required to obtain definite conclusion for population history in KNP. The *Ne* of LZNP, MGMA, and NWR were relatively small. NWR had the smallest *Ne* at 32.1 with a 95% confidence interval of 24.3–45.5 (Table 5). This *Ne* estimate is lower than any *Ne* estimate observed in tsetse flies in the same Morsitans group tsetse fly, *G*. *pallidipes*. Estimated *Ne* using microsatellites were 180 and 551 in different regions of Kenya [54]. The low *Ne* of NWR and the restricted gene flow between NWR and the other locations indicate that NWR is potentially an isolated location with relatively small population size. Therefore, cost-effective tsetse control activities may be possible. However, the evidence presented in this study is not strong enough to definitely draw this conclusion, since the other locations surrounding Lake Malawi have not been explored in this study. In addition, the *Ne* estimates should be handled with care, since the census population densities are usually much greater than the estimated *Ne*, and the *Ne* estimation is affected by a number of demographic and genetic phenomena, such as sex ratio and temporal variation in population size [55]. In this study, the estimation of *Ne* was conducted using both female and male flies. Although care was taken to make the sex ratio 50:50, we cannot rule out the possibility of the biasing affect leading to smaller *Ne* estimates.

### Conclusions

We analyzed partial mitochondrial CO1 sequences and 10 microsatellite loci of *G*. *m*. *morsitans* collected from three locations of Zambia and two locations from Malawi, and identified two genetically separated clusters: NWR and others. This result was in keeping with previous descriptions of the distribution of *G*. *morsitans morsitans* in East and Southern Africa. There appears to be restricted gene flow between NWR and the other locations, and we hypothesize that the escarpment of the Great Rift Valley acts as an environmental barrier, since its high altitude is at the limit of the tsetse fly’s biological habitat range. In addition to its apparently restricted gene flow, the small effective population size indicates that NWR may be a population where tsetse control activities can be applied at a lower cost compared to non-isolated populations [56]. Tsetse control activities include artificial baits, insecticide-treated cattle (ITC), aerial spraying, and the sterile insect technique (SIT) in combination with the insecticide-based methods. The Restricted Application Protocol (RAP) using ITC has been shown to be the most cost-effective control method in areas where tsetse flies and livestock co-exist [57]. However, further research will be needed to identify the genetic population structure in other low-altitude sites around Lake Malawi that have not been included in this study, in order to confirm that there are no re-invasions into NWR from adjacent areas. We have detected a new location (KNP) infested by *G*. *m*. *morsitans*, which has not been illustrated in the previous *G*. *m*. *morsitans* distribution maps. KNP is probably a part of the major tsetse belt in the Luangwa river basin, and has a relatively large effective population size. Further study is needed to elucidate the extent of the tsetse belt, and assess the migration into adjacent reservoir communities.

## Supporting information

S1 Fig Mismatch distribution of pairwise differences. The X-axis represents the number of pairwise differences, and the Y-axis represents their frequency.
(TIF)

S2 Fig Delta K plot for detecting the most likely K for *Glossina morsitans morsitans* individuals from five locations. Plots were generated using the Evanno method [44] implemented in STRUCTURE HARVESTER v0.6.94.
(TIF)

S1 Table Haplotype distributions among each *Glossina morsitans morsitans* populations. (XLSX)

S2 Table Linkage disequilibrium *p*-value for each locus pair across all populations. (XLSX)
