# Supplementary figures and images for: Genetic diversity and population structure of Glossina morsitans morsitans in the active foci of human African trypanosomiasis in Zambia and Malawi
Source: PLoS Negl Trop Dis. 2019 Jul 25;13(7):e0007568. doi: 10.1371/journal.pntd.0007568 (PMC6657825; doi:10.1371/journal.pntd.0007568)

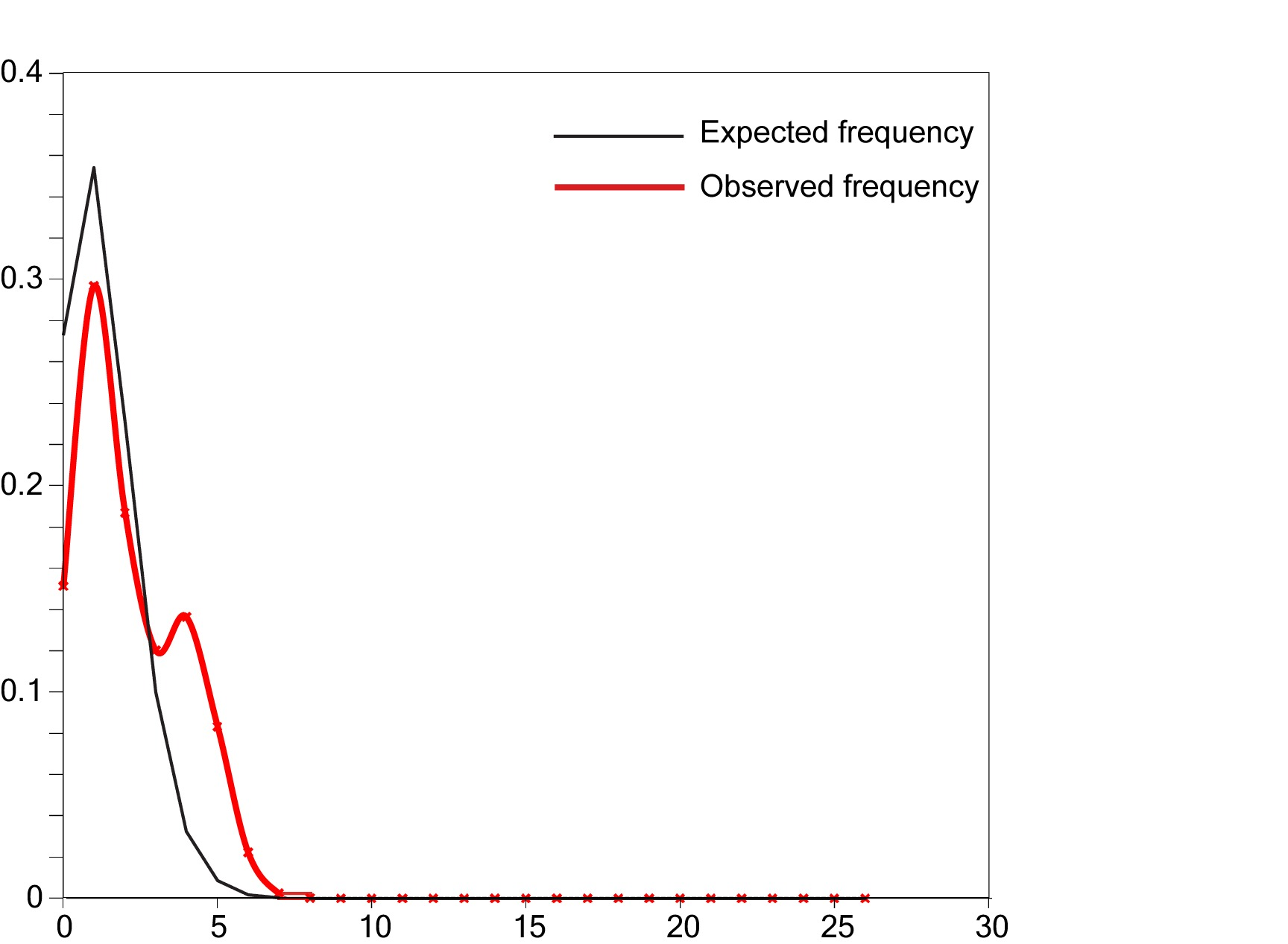

Supplement: S1 Fig — The X-axis represents the number of pairwise differences, and the Y-axis represents their frequency. (TIF) [file pntd.0007568.s001.tif]

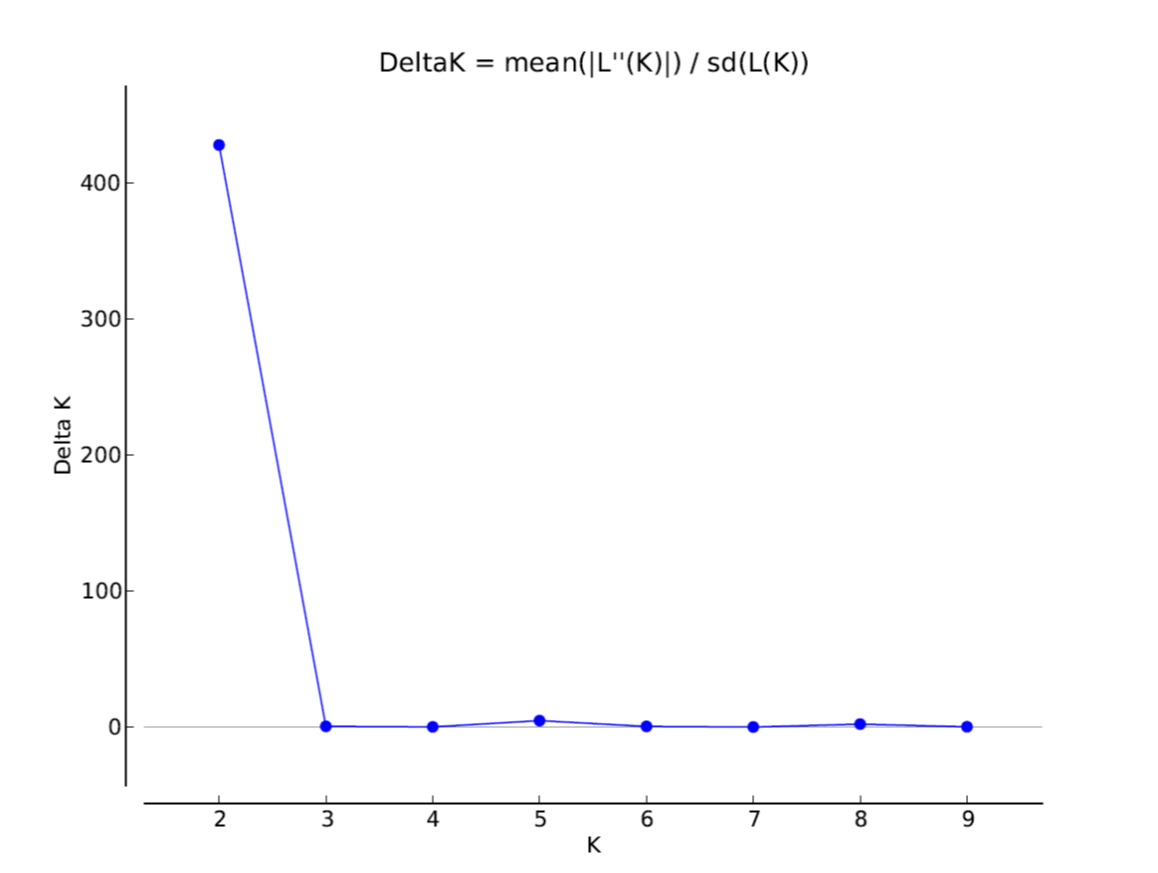

Supplement: S2 Fig — Plots were generated using the Evanno method [44] implemented in STRUCTURE HARVESTER v0.6.94. (TIF) [file pntd.0007568.s002.tif]
